# Longitudinal network analysis of mental health trends in Chinese university freshmen: a decadal study (2014–2023)

**DOI:** 10.3389/fpsyg.2025.1611264

**Published:** 2025-09-25

**Authors:** Xiujie Teng, Xiaoyan Wang, Ye Liu, Dingchao Wu, Zhenxuan Dong, Shaobo Lyu

**Affiliations:** ^1^Capital University of Economics and Business, Beijing, China; ^2^Library, North China University of Science and Technology, Tangshan, Hebei, China; ^3^School of Psychology and Mental Health, Hebei Key Laboratory of Mental Health and Brain Science, Tangshan, Hebei, China

**Keywords:** freshmen, network analysis, SCL-90, time series, mental health

## Abstract

**Introduction:**

Global mental health deterioration among young adults is a growing concern. This study aims to map the psychopathological architecture of mental health symptoms in a large cohort of university freshmen to identify core symptoms that could be pivotal for intervention strategies.

**Methods:**

A network analysis was conducted on annual mental health assessments of 24,047 university freshmen over a ten-year period (2014–2023). Symptoms were assessed using the Symptom Checklist-90 (SCL-90).

**Results:**

The mental health symptom network structure demonstrated remarkable stability across the decade. Anxiety, depression, and interpersonal sensitivity were consistently identified as the most central symptoms within the network. The rank order of symptoms by nodal strength was: anxiety, depression, interpersonal sensitivity, psychoticism, obsessive symptoms, paranoia, hostility, phobia, and somatization.

**Discussion:**

The findings highlight anxiety, depression, and interpersonal sensitivity as prominent and central psychological challenges for college students. The stability of this network structure suggests these core symptoms are reliable targets for priority screening and the development of targeted mental health interventions and preventative treatments within university populations.

## 1 Introduction

In recent years, the mental health of college students has become a social problem faced by countries around the world, which has attracted significant attention from governments around the world (Song et al., 2024). In particular, the sudden outbreak and rapid spread of the COVID-19 epidemic in the world has led to the widespread development of various mental disorders among college students (Zhang et al., 2024a). A national study in the United States showed that more than 60% of American college students met the criteria for one or more mental health problems during the period of concentrated outbreaks (2020–2021). This represents a significant increase from about 50 per cent in 2013 (Lipson et al., 2022). Meanwhile, more than 40 per cent of French university students reported having at least one psychological severe problem, and 12.4 per cent had sought help from a professional mental health worker (wallet et al.,2020). A study of mental health and intervention policies among Chinese college students showed that the prevalence of depression and anxiety among Chinese college students was as high as 37.4 and 19% (Yang et al., 2023b), and sleep disturbances and stress continued throughout college (Gardani et al., 2022).

As a unique demographic group typically aged just over 18, college students frequently encounter adaptation barriers stemming from shifts in identity, psychological states, learning styles, and social networks. Anxiety and depression have emerged as predominant mental health issues among this population, with notable increases in the prevalence of insomnia, obsessive-compulsive behaviors, and suicidal ideation (Zarowski et al., 2024). In conclusion, understanding the status of college students’ mental health and analyzing the relationships between their psychological symptoms are crucial for public health initiatives.

First-year undergraduates are particularly vulnerable to multidimensional stressors during environmental transitions (Liu et al., 2023). Most students leave home independently for the first time, resulting in a lack of emotional support and potential communication barriers due to geographical and cultural differences. These challenges may further lead to social anxiety and interpersonal conflicts. Financial strain also plays a critical role, especially for students from low-income families, as high tuition fees and living expenses can create significant pressure, negatively impacting their academic performance and daily life. Therefore, understanding the psychological challenges faced by students and implementing targeted interventions are imperative. The mental health status of college students is trending in an increasingly concerning direction (Ma et al., 2020), necessitating urgent societal attention and coordinated efforts.

Existing research on college students’ mental health has predominantly focused on symptom prevalence rates and risk/protective factor identification (Li et al., 2022). However, few studies systematically examine symptom-to-symptom interactions, which could reveal critical pathways for targeted interventions (Pan et al., 2024).

Network analysis, an emerging methodology integrating multivariate statistics and network science, provides a paradigm shift for modeling psychological constructs (Khan et al., 2018). This approach conceptualizes mental health symptoms as interconnected nodes in a dynamic system, where statistical dependencies (e.g., partial correlations) define edge weights between nodes (Pan et al., 2024). Unlike latent variable models that assume a common underlying cause, network analysis explicitly maps symptom-level interactions (Borsboom et al., 2021). Its graphical output enables intuitive visualization of complex dependency patterns, making it particularly effective for exploratory research with limited theoretical priors.

Many studies have studied the connection between SCL-90 symptoms through network analysis. For example, the network analysis of patients with eating disorders shows that the bridge core symptoms related to the pursuit of slimness are anxiety, depression and interpersonal sensitivity (Solmi et al., 2018); In the study of the mechanism of mental health in physical exercise, anxiety, depression, and interpersonal sensitivity have been proven to be the core symptoms of ball sports and nocturnal activities (Yang et al., 2024). While prior studies (Wang et al., 2024; Zhang et al., 2024b; Zhang et al., 2024c) have mapped the nine-dimensional symptom network of the SCL-90, their reliance on cross-sectional data precludes a comprehensive understanding of temporal symptom dynamics. This study explores the network of the symptom network of SCL-90 in freshmen, utilizing a decade-long longitudinal dataset.

## 2 Participants

The data were collected in an ongoing longitudinal study of SCL-90 data from 2014 to 2023 in Beijing, China, from 2014 to 2023. The demographic characteristics of the study sample are shown in Table 1, including gender and age information. The diversity of participants at each point in time is largely due to a weak change in the school’s admissions program. All data are collected in October of each year. This study has been approved by the Ethics Committee of North China University of Science and Technology (Approval no. 13065).

**Table 1:** Ten-year demographic description.

Year	2014	2015	2016	2017	2018	2019	2020	2021	2022	2023	Totals
Totals	2,431	2,344	2,399	2,365	2,442	2,389	2,325	2,341	2,394	2,616	24,046
Male	774	764	829	800	828	938	861	981	901	1,041	8,717
Female	1,657	1,580	1,570	1,565	1,614	1,451	1,464	1,360	1,493	1,575	15,329
Average age	Missing	Missing	18.16	18.15	18.30	18.49	18.20	18.47	19.26	18.36	19.79
Standard deviation of age	Missing	Missing	0.77	0.95	1.56	2.08	1.11	0.97	0.76	0.69	

## 3 Measures

### 3.1 The symptom checklist 90

The Symptom Checklist 90 (SCL-90) (Derogatis and Cleary, 1977) was used to collected data, which contains a total of 90 item questions. The 90 items can be classified into 10 factors including somatization, obsessive-compulsive symptoms, interpersonal sensitivity, depression, anxiety, hostility, fear, paranoia, psychoticism and other items. Each item is scored using a 5-level scoring system. This table is currently the most widely used outpatient examination scale for psychiatric disorders and mental disorders. SCL-90 scale has become a tool for preliminary evaluation and screening of mental health in colleges and universities. At the beginning of each academic year, schools will organize colleges to complete online scale to test students’ mental health level. The SCL-90, as a classic multidimensional symptom checklist, covers nine factors including somatization, obsessive-compulsive symptoms, interpersonal sensitivity, depression, and anxiety, enabling a comprehensive capture of various psychological symptoms that may arise in adolescents during school adaptation. Its ‘long-form’ design aligns well with the study’s need to analyse ‘complex symptom networks,’ enabling more precise identification of association patterns between different symptoms (e.g., the co-occurrence of anxiety and depression, or the interactive influence between obsessive-compulsive symptoms and interpersonal sensitivity). The SCL-90’s multidimensional design avoids potential information gaps that may arise from shorter scales, providing a more robust data foundation for subsequent analyses of ‘symptom development trajectories.’ Although short scales are currently more favored due to their convenience, the SCL-90 has been validated through years of clinical and research use, demonstrating good reliability and validity. Additionally, its normative data are abundant, facilitating cross-comparisons with results from similar adolescent studies, making it particularly suitable for the core objective of this study: a ‘large-scale baseline survey of symptom characteristics in a population’. we conducted annual assessments of the SCL-90’s reliability and validity to ensure consistent measurement quality. The specific results are as follows. The Cronbach’s alpha coefficients for each year of the scale are shown in Supplementary Table S2.

### 3.2 Network structure diagram

A correlation matrix of the 10 symptom dimensions was constructed using the Pearson correlation coefficient. The partial correlation network structure was then visualized using the qgraph package, which was employed to create a more concise network representation. In estimating correlation networks, we retained the default threshold of 0 in the qgraph; edges whose absolute edge-weights did not exceed this value were automatically dropped from the model. To enhance visual interpretability, we subsequently set minimum = 0.10, thereby suppressing the display of edges whose absolute weights were below 0.10. In the network, each node represents a symptom dimension. In the graphical output, the positioning of the nodes reflects their centrality within the network, and the distance between nodes indicates the strength of their association. Edge thickness (line thickness) represents the intensity of the correlation, with blue borders indicating positive correlations and red borders indicating negative correlations. All analyses were conducted using R software (qgraph and bootnet).

### 3.3 Centrality indices

Two node centrality indices were calculated to quantify the structural importance of each node within the network (Proctor and Loomis, 1951), specifically strength and closeness. Strength denotes the direct connection between a node and other nodes, while closeness is defined as the inverse of the sum of the shortest distances from a given node to all other connected nodes. These indices were reported as standardized values (i.e., z-scores), where higher values indicate greater centrality of a node within the network. Betweenness, which measures the extent to which a node lies on the shortest paths between pairs of nodes, is another important centrality measure. However, betweenness is considered less relevant in psychological networks and has been found to be less stable overall (Seeley, 1949).

### 3.4 Testing for significant differences

The Network Comparison Test package can, in principle, evaluate differences across the 10 annual networks. We therefore conducted two invariance tests—global strength invariance and edge-wise invariance. However, any sequence of pairwise year-to-year comparisons would necessitate a large number of significance tests. After Bonferroni correction (Bland and Altman, 1995), the adjusted *α*-level would be approximately 0.0011. Under this stringent threshold, none of the observed *p*-values reached significance.

Furthermore, the NCT relies on bootstrap difference tests, whose statistical power is generally modest. Such tests are prone to under-detect true population-level differences, so non-significant centrality indices should not be interpreted as evidence that centralities are equal or that they have been estimated with insufficient accuracy (Epskamp et al., 2018). Consequently, we advise caution when interpreting the outcomes of bootstrap-based difference tests. The results are shown in Table S3 and Table S4 in the Supplementary materials.

## 4 Results

According to the results of the SCL-90 questionnaire, 10 psychological dimensions were obtained: somatization, obsessive-compulsive symptoms, interpersonal sensitivity, depression, anxiety, hostility, terror, paranoia, and psychosis. The scoring criteria of the corresponding items were summarized, and the weight rules were applied to determine the scores of each dimension.

To distinguish these psychological dimensions when constructing the network model, we divided these psychological dimensions into: Somatization as ‘So’; ‘Ob’ stands for obsessive symptoms; ‘Ip’ stands for interpersonal sensitivity; ‘De’ stands for depression; ‘An’ stands for anxiety; ‘Ho’ stands for hostility; ‘Ph’ stands for phobia; ‘Pa’ stands for paranoia; ‘Ps’ stands for psychoticism; and ‘Ot’ stands for other.

### 4.1 Data stability check

The stability of the data was assessed using the R 4.2.2 package (bootnet). The analysis results revealed that the stability coefficient exceeded 0.7 in each study year. A coefficient of CS = 0.70 indicates the maximum acceptable level of sample reduction, with values above 0.50 considered acceptable, and a minimum threshold of 0.25 (Epskamp et al., 2018). To assess the stability of these retained edges, we followed recommended practice (Epskamp et al., 2018) and computed 1,000 non-parametric bootstrap samples using the bootnet function with the specification bootnet (type = “case,” nBoots = 2000). Correspondingly, Figure S1-S10 illustrates that the centrality indices of strength and closeness demonstrated good stability. These findings demonstrate a high level of stability, supporting the robustness of the subsequent analyses.

### 4.2 Ten-year network structure analysis

**Figure 1:**
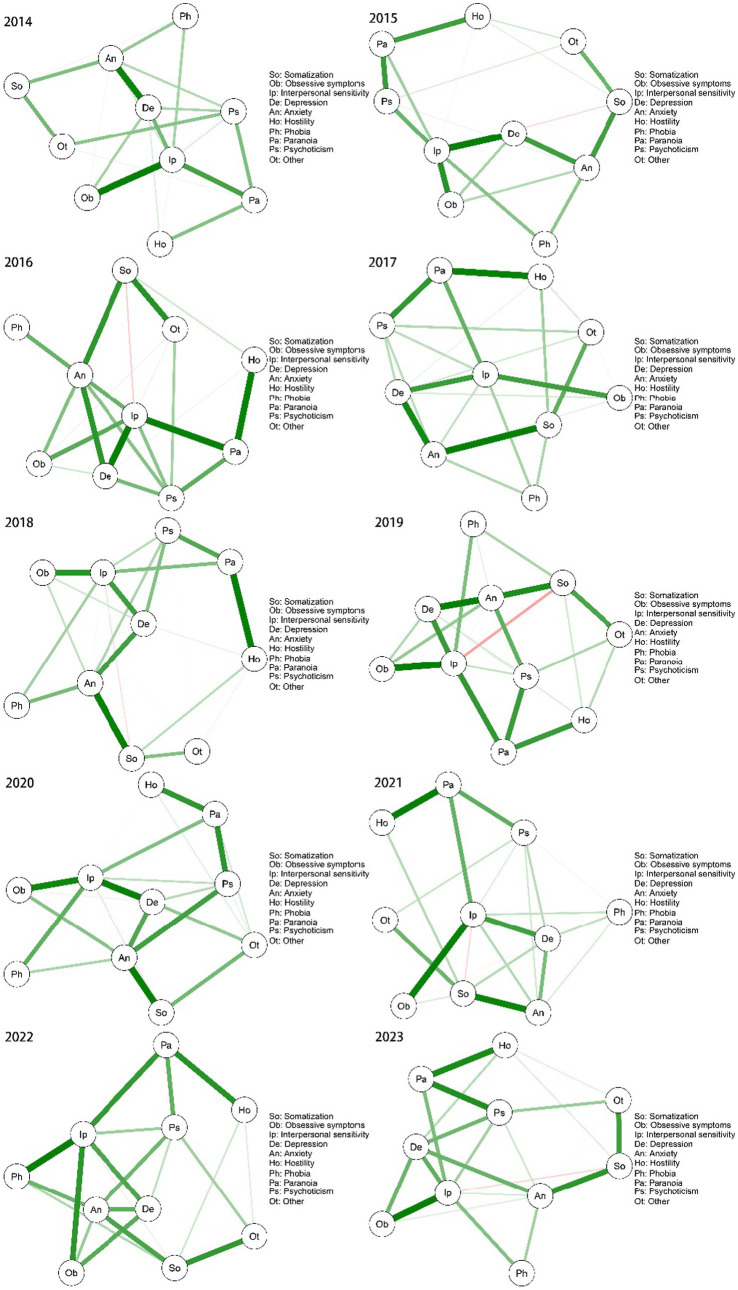
Network structure of the 10-year symptom. Circles indicate different nodes. The side line shows the correlation between the two, the thickness of the line indicates the strength of the correlation, green indicates a positive correlation, and red indicates a negative correlation. (Same as below).

**Figure 2:**
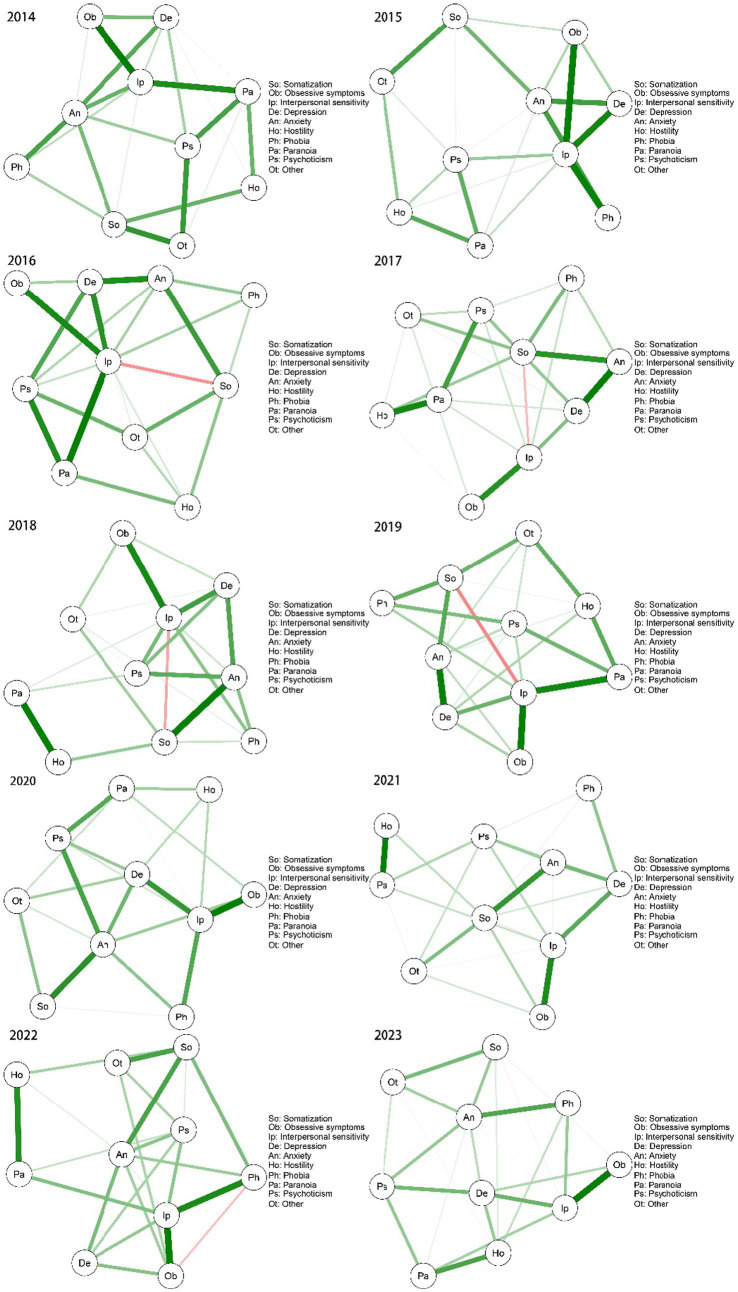
Network structure of the male decade symptoms.

**Figure 3:**
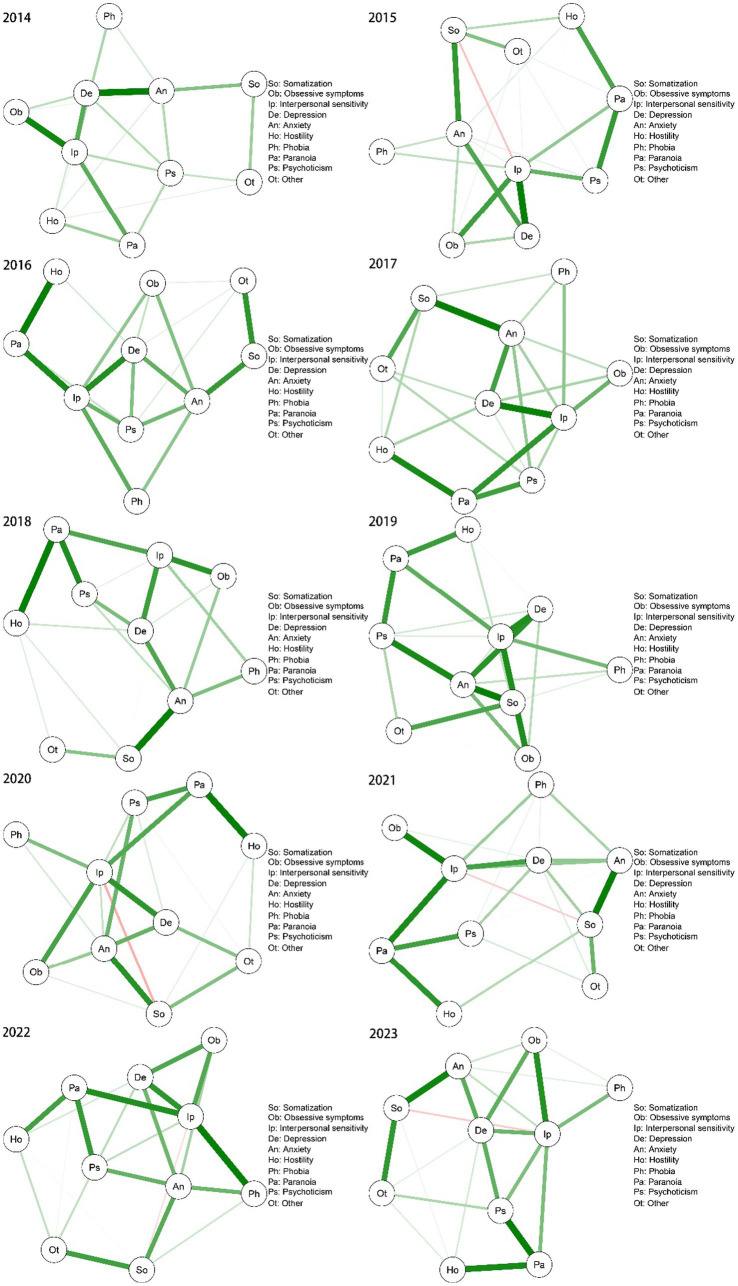
Network structure of symptoms in the female decade.

To gain an understanding of the mental health landscape among college students, we used the qgraph package in R 4.2.2 to process the data and construct a network diagram of mental health over a 10-year period (Figure 1). By grouping the samples by gender, we mapped the network structure for males (Figure 2) and females (Figure 3) separately to show how gender makes a difference in the network structure. There were significant differences in node correlations between males and females across years, which may be related to changes in social context or behavioral patterns. The analysis indicated that the overall network structure remained stable, with certain nodes, such as “interpersonal sensitivity,” “depression,” and “anxiety,” consistently occupying central positions. While the correlations between nodes remained largely consistent, the strength of these correlations varied significantly across years. Additionally, a persistent, weak negative relationship was observed between somatization and interpersonal sensitivity.

### 4.3 Node centrality index

**Figure 4:**
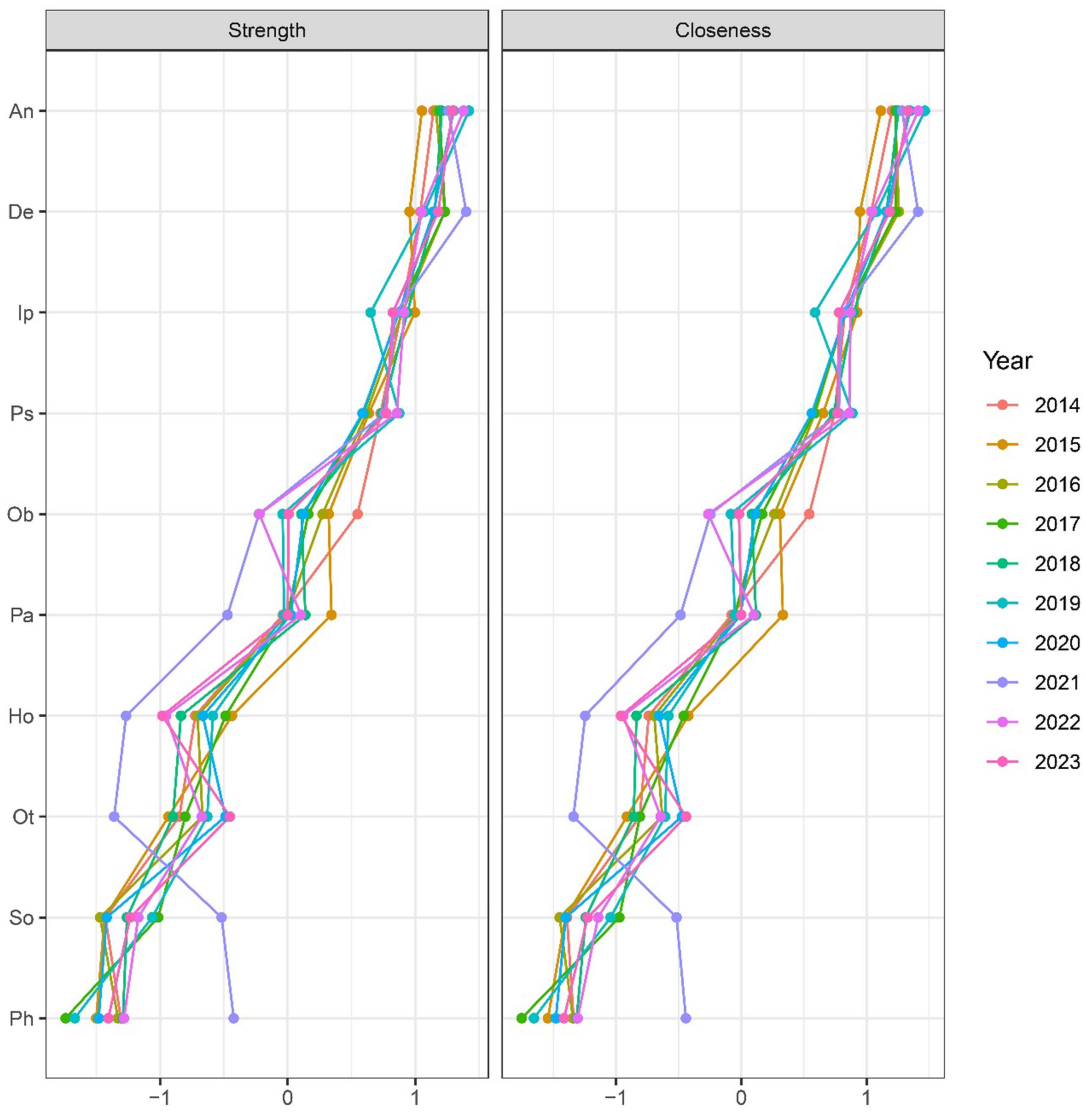
Line plots of node intensity ranked by year and summarized. n: anxiety; De: depression; Ip: interpersonal sensitivity; Ps: psychoticism; Ob: obsessive symptoms; Ph: phobia; Ho: hostility; Ot: others; So: somatization; Pa: paranoia.

To identify the central symptoms of college students’ mental health, we employed the R 4.2.2 package (qgraph:centralityPlot) to generate and summarize 10 line graphs, sorted by node strength for each year (Figure 4). The centrality coefficient was represented by standardized Z-scores, with node strength reflecting the sum of the absolute values of the correlation coefficients between two nodes. The qgraph:centrality_auto function was used to compute the centrality index for each node over the 10-year period. The results indicated that the centrality indices for anxiety, depression, and interpersonal sensitivity remained consistently high. Additionally, the strength of symptoms related to terror and somatization increased significantly in 2021, while paranoid ideation and hostility showed a notable decrease. These changes may be associated with the impacts of the COVID-19 pandemic.

## 5 Discussion

To examine the trend in mental health status and identify the core symptoms of first-year students, we analyzed the SCL-90 measurement data using network analysis over a 10-year period. Our findings suggest that, despite temporal variations, certain central symptoms—namely anxiety, depression, and interpersonal sensitivity—remained consistently at the core of college students’ mental health. Furthermore, symptoms such as somatization, paranoia, phobia, and hostility appeared to be more sensitive to external stressors, including the COVID-19 pandemic.

### 5.1 The network structure and central nodes are stable

We constructed a 10-year network structure (Figure 1) based on the data and found that the overall network structure remained stable over the decade. Anxiety, depression, and interpersonal sensitivity consistently occupied central positions in the network. According to the 2022 Survey on the Mental Health of Chinese College Students, the overall mental health of college students was reported as generally good, but a considerable proportion of students remained at risk for anxiety and depression. Specifically, 78.52% of students reported no risk for depression, while only 54.72% were free of anxiety risk (Ooi et al., 2022). A separate study examining anxiety, depression, and interpersonal relationships found a significant positive correlation between anxiety, depression, and interpersonal distress among college students, suggesting that these mental health issues may exacerbate one another, leading to heightened psychological distress (Jacobson and Anderson, 1982; Liu et al., 2022).

First-year students are particularly vulnerable to adjustment disorders as they transition to university life. Previous research indicates that first-year students face challenges in academic performance, interpersonal communication, and role adaptation, which can lead to symptoms such as anxiety, loss of appetite, and insomnia. In severe cases, these issues may result in impulsive behaviors like dropping out or self-harm. Physical illnesses and stress exceeding certain thresholds can also trigger psychological problems. The development of freshman adjustment disorder is influenced by social, historical, cultural, and personal factors, such as the pressure of exam-oriented education, a lack of transition support for first-year students, family environment, and individual psychological resilience (Cai and Wu, 2016). Coping strategies employed in stressful situations represent a core mechanism in the psychological pathology underlying the onset and progression of anxiety and depression (Lin et al., 2011; Ramadianto et al., 2022; Seighali et al., 2024; Serrano et al., 2021). Defined as cognitive and behavioral efforts to manage stressors—specifically, to control, reduce, or tolerate distress arising from interpersonal and environmental demands—these strategies broadly encompass problem-focused and emotion-focused approaches (Folkman, 2013; Suls and Fletcher, 1985) Therefore, it is crucial to choose an appropriate response to deal with the pressure you face. Given the significant barriers first-year students face, it is essential to implement strategies that bridge the gap between high school and university life to better support students’ transitions (Huang and Li, 2010). Therefore, it is crucial to strengthen mental health monitoring, provide early intervention, and promote both physical and mental wellbeing.

### 5.2 Node centrality index

To further investigate the central symptoms, we standardized and ranked the centrality indices. Anxiety, depression, and interpersonal sensitivity consistently ranked as the most prominent symptoms of psychological abnormalities in college students. The 2022 survey on the mental health of Chinese college students highlighted that lifestyle factors—such as sleep problems, stress, and boredom—significantly impact students’ mental health. Poor sleep quality was particularly associated with a higher risk of depression and anxiety, while elevated stress and boredom also correlated with increased mental health risks (Doane et al., 2015; Nyer et al., 2013).

Interpersonal sensitivity has been identified as a risk factor for several psychological issues, including depression, anxiety, and loneliness. Students with high interpersonal sensitivity tend to experience low self-esteem and social anxiety, which may reduce social engagement and increase feelings of isolation. Research suggests that such students are more prone to loneliness due to a lack of social support (Moeller and Seehuus, 2019; Yan, 2007; Yang et al., 2023a). Cacioppo’s Evolutionary Theory of Loneliness (ETL) posits that loneliness functions as an aversive warning signal for perceived social isolation (Cacioppo and Cacioppo, 2018). This mechanism directs attention to potential deteriorations in physical wellbeing, thereby motivating individuals to seek social engagement. Such reparative behaviors ultimately restore beneficial relationships, promoting adaptive outcomes essential for long-term evolutionary fitness. Therefore, it is necessary to pay close attention to students’ mental health and help them improve their social adaptability. These findings indicate that the psychological status of first-year students entering university is far from optimistic. Without prompt intervention, these symptoms may escalate, resulting in a vicious cycle of psychological distress (Beiter et al., 2015; Li et al., 2022).

Additionally, we observed a significant increase in the centrality of somatization in 2021, which may be linked to the COVID-19 pandemic. The widespread infection and its associated physical health impacts could have contributed to heightened somatic symptoms, as reflected in the SCL-90. This rise in somatization is consistent with findings from other studies that report heightened somatic symptoms during times of health anxiety, such as during the pandemic. This trend underscores the influence of major social events on college students’ mental health, particularly for first-year students who are already prone to adjustment issues (Chaturvedi, 2020). The psychological effects of such events are more pronounced among students in this transitional phase.

Considering this phenomenon, research indicates the COVID-19 pandemic significantly exacerbated mental health challenges among first-year college students. Studies observed increased levels of psychological distress, including anxiety and depression, during this period compared to pre-pandemic baselines (Copeland et al., 2021; Wang et al., 2023). Academic disruptions and health-related uncertainties were identified as prominent stressors contributing to this decline. Furthermore, heightened physical manifestations of distress, such as somatic symptoms, were documented in this population, particularly among those experiencing greater academic and lifestyle instability. The pervasive uncertainty surrounding the pandemic was also found to intensify feelings of helplessness and anxiety, especially impacting students navigating the transition to college life. These findings collectively illustrate the pandemic’s multifaceted adverse impact on first-year students’ mental wellbeing, with somatization representing a significant component of this response (Cao et al., 2020).

### 5.3 Limitations and future research directions

Strengths of this study include its innovative application of network analysis, which transcends traditional latent variable approaches by mapping dynamic symptom interactions, and its decade-long design that captures temporal trends and external event impacts (e.g., COVID-19). The use of centrality indices (strength, closeness) provided granular insights into symptom hierarchies, enhancing clinical prioritization.

Limitations include reliance on self-reported SCL-90 data, which may introduce response bias, and the scale’s focus on symptom severity rather than contextual or protective factors (e.g., social support). Additionally, the study did not explore gender or cultural differences in symptom networks, limiting generalizability.

The approximately 20,000 freshmen included in this study share certain homogeneous characteristics as they are all university students who enrolled in the same academic year. These characteristics primarily manifest in the following aspects: age concentration within the 18–20 age range, similar educational backgrounds (all graduated from regular high schools), and a similar transitional state from family to campus living environments. This homogeneity reduces the interference of demographic variables on the core indicators of the study, facilitating a focus on developmental issues shared by the group. However, it may also limit the generalizability of the study results to broader populations. Therefore, the findings of this study are only applicable to a similar group of university freshmen and cannot be generalized to more widespread populations.

It should be noted that this study is an observational study based on routine admission health records and is not a clinical intervention study. Due to constraints from the original research design and ethical guidelines (large-scale screening without clear medical indications may increase students’ psychological burden), standardized psychiatric screening scales were not included. As a result, there may be unidentified potential mental health issues in the sample, such as subclinical depression, anxiety, or other untreated psychological disorders. These unobserved variables may have a potential impact on indicators like ‘student adaptation status’ and ‘emotional regulation ability’ in the study. For example, undiagnosed potential disorders may lead to higher adaptation difficulty scores among some students, but the proportion and extent of such individuals’ impact cannot be precisely quantified using the existing data.

Future research could collaborate with clinical partners to conduct targeted mental health screenings on large non-clinical populations within ethical boundaries. This would enable more precise differentiation between ‘developmental adaptation issues’ and ‘pathological psychological symptoms’.

Inconsistencies in population statistics reports are a significant limitation of this study. The missing age and standard deviation data from the early stages of the study (2014–2015) represent an objective data gap caused by incomplete data collection, not intentional omission. During this phase, which was the initial stage of the study, the data collection process had not yet been fully standardized. Some field research sites failed to record age information completely due to operational oversights, resulting in the discovery of missing data during subsequent data. We have verified the original survey records and confirmed that no backup copies of the missing data exist, making it impossible to complete the data through supplementation. After calculation, the 2014–2015 data account for 19.85% of the total sample size, with the missing variable being age. However, the core analytical indicator of this study is the node centrality index, and the absence of age data has minimal impact on this indicator and the overall time series trend (as data from subsequent years are complete and the sample size is sufficient).

## Data Availability

The raw data supporting the conclusions of this article will be made available by the authors, without undue reservation.
