# Supplementary material for: Longitudinal network analysis of mental health trends in Chinese university freshmen: a decadal study (2014–2023)
Source: Front Psychol. 2025 Sep 25;16:1611264. doi: 10.3389/fpsyg.2025.1611264 (PMC12509066; doi:10.3389/fpsyg.2025.1611264)

# Supplementary Materials

Table

Table 1 Ten-year demographic description

|  | 2014 | 2015 | 2016 | 2017 | 2018 | 2019 | 2020 | 2021 | 2022 | 2023 | totals |
| --- | --- | --- | --- | --- | --- | --- | --- | --- | --- | --- | --- |
| totals | 2431 | 2344 | 2399 | 2365 | 2442 | 2389 | 2325 | 2341 | 2394 | 2616 | 24046 |
| male | 774 | 764 | 829 | 800 | 828 | 938 | 861 | 981 | 901 | 1041 | 8717 |
| female | 1657 | 1580 | 1570 | 1565 | 1614 | 1451 | 1464 | 1360 | 1493 | 1575 | 15329 |
| Average age | missing | missing | 18.16 | 18.15 | 18.30 | 18.49 | 18.20 | 18.47 | 19.26 | 18.36 | 19.79 |
| Standard deviation of age | missing | missing | 0.77 | 0.95 | 1.56 | 2.08 | 1.11 | 0.97 | 0.76 | 0.69 |  |

**Table S2** Cronbach's alpha coefficients for each year of the scale

| **year** | **2014** | **2015** | **2016** | **2017** | **2018** | **2019** | **2020** | **2021** | **2022** | **2023** |
| --- | --- | --- | --- | --- | --- | --- | --- | --- | --- | --- |
| **SCL-90** | 0.97 | 0.97 | 0.97 | missing | 0.98 | 0.98 | 0.98 | 0.98 | 0.98 | 0.98 |
| **Somalization** | 0.82 | 0.83 | 0.83 | missing | 0.86 | 0.87 | 0.86 | 0.88 | 0.89 | 0.88 |
| **Obsessive symptoms** | 0.83 | 0.83 | 0.84 | missing | 0.85 | 0.85 | 0.85 | 0.87 | 0.89 | 0.89 |
| **Interpersonal sensitivity** | 0.82 | 0.83 | 0.85 | missing | 0.85 | 0.85 | 0.85 | 0.87 | 0.87 | 0.87 |
| **Depression** | 0.88 | 0.87 | 0.89 | missing | 0.90 | 0.91 | 0.91 | 0.91 | 0.91 | 0.92 |
| **Anxiety** | 0.83 | 0.82 | 0.84 | missing | 0.86 | 0.87 | 0.86 | 0.88 | 0.88 | 0.88 |
| **Hostility** | 0.76 | 0.76 | 0.76 | missing | 0.79 | 0.83 | 0.82 | 0.82 | 0.82 | 0.83 |
| **Phobia** | 0.71 | 0.67 | 0.72 | missing | 0.75 | 0.74 | 0.74 | 0.81 | 0.81 | 0.79 |
| **Paranoia** | 0.72 | 0.74 | 0.74 | missing | 0.76 | 0.79 | 0.78 | 0.81 | 0.80 | 0.81 |
| **Psychoticism** | 0.77 | 0.77 | 0.79 | missing | 0.79 | 0.81 | 0.81 | 0.84 | 0.84 | 0.84 |
| **Other** | 0.64 | 0.65 | 0.64 | missing | 0.69 | 0.72 | 0.69 | 0.77 | 0.76 | 0.78 |

**Table S3** ten years global strength invariance

|  | **2014** | **2015** | **2016** | **2017** | **2018** | **2019** | **2020** | **2021** | **2022** | **2023** |
| --- | --- | --- | --- | --- | --- | --- | --- | --- | --- | --- |
| **2014** |  |  |  |  |  |  |  |  |  |  |
| **2015** | 0.48 |  |  |  |  |  |  |  |  |  |
| **2016** | 0.78 | 0.78 |  |  |  |  |  |  |  |  |
| **2017** | 0.22 | 0.56 | 0.30 |  |  |  |  |  |  |  |
| **2018** | 0.37 | 0.82 | 0.60 | 0.53 |  |  |  |  |  |  |
| **2019** | 0.11 | 0.42 | 0.19 | 0.82 | 0.46 |  |  |  |  |  |
| **2020** | 0.72 | 0.71 | 0.96 | 0.32 | 0.58 | 0.11 |  |  |  |  |
| **2021** | 0.04 | 0.01 | 0.02 | 0.21 | 0.01 | 0.25 | 0.03 |  |  |  |
| **2022** | 0.21 | 0.55 | 0.33 | 0.79 | 0.76 | 0.65 | 0.29 | 0.11 |  |  |
| **2023** | 0.09 | 0.52 | 0.18 | 0.84 | 0.60 | 0.73 | 0.30 | 0.09 | 0.92 |  |

**Table S4 year-to-year edge-wise invariance**

|  | **2014** | **2015** | **2016** | **2017** | **2018** | **2019** | **2020** | **2021** | **2022** | **2023** |
| --- | --- | --- | --- | --- | --- | --- | --- | --- | --- | --- |
| **2014** |  |  |  |  |  |  |  |  |  |  |
| **2015** | 0.37 |  |  |  |  |  |  |  |  |  |
| **2016** | 0.62 | 0.09 |  |  |  |  |  |  |  |  |
| **2017** | 0.76 | 0.24 | 0.69 |  |  |  |  |  |  |  |
| **2018** | 0.35 | 0.04 | 0.81 | 0.65 |  |  |  |  |  |  |
| **2019** | 0.89 | 0.16 | 0.62 | 0.73 | 0.71 |  |  |  |  |  |
| **2020** | 0.060 | 0.14 | 0.52 | 0.13 | 0.36 | 0.31 |  |  |  |  |
| **2021** | 0.05 | 0.09 | 0.15 | 0.01 | 0.02 | 0.11 | 0.20 |  |  |  |
| **2022** | 0.14 | 0.55 | 0.69 | 0.06 | 0.27 | 0.40 | 0.44 | 0.05 |  |  |
| **2023** | 0.82 | 0.06 | 0.71 | 0.28 | 0.81 | 0.22 | 0.50 | 0.05 | 0.55 |  |

**Figure S1-S10 Regarding the stability results (CS) of the ten-years networks**

Average correlations between centrality indices of networks sampled with persons dropped and the original sample. Lines indicate the means, and areas indicate the range from the 2.5th quantile to the 97.5th quantile.
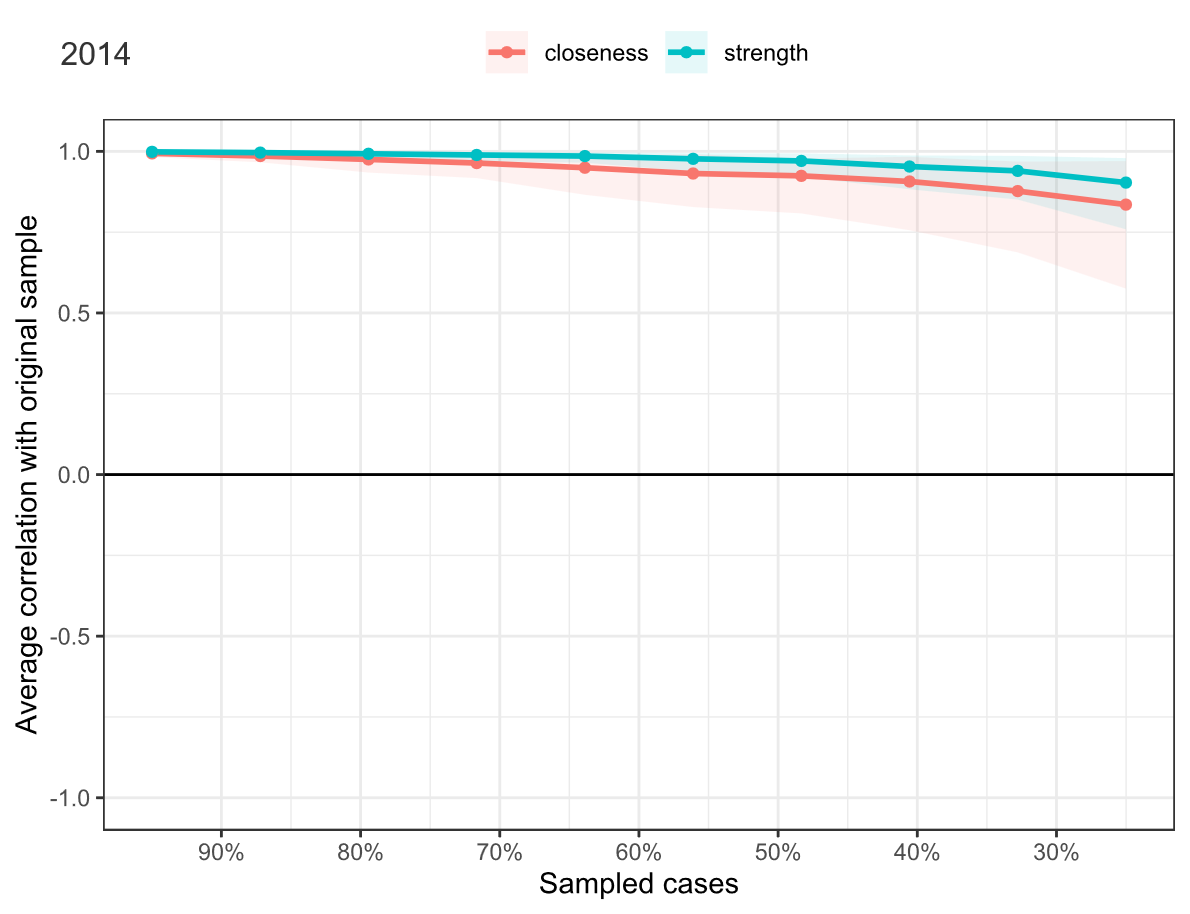

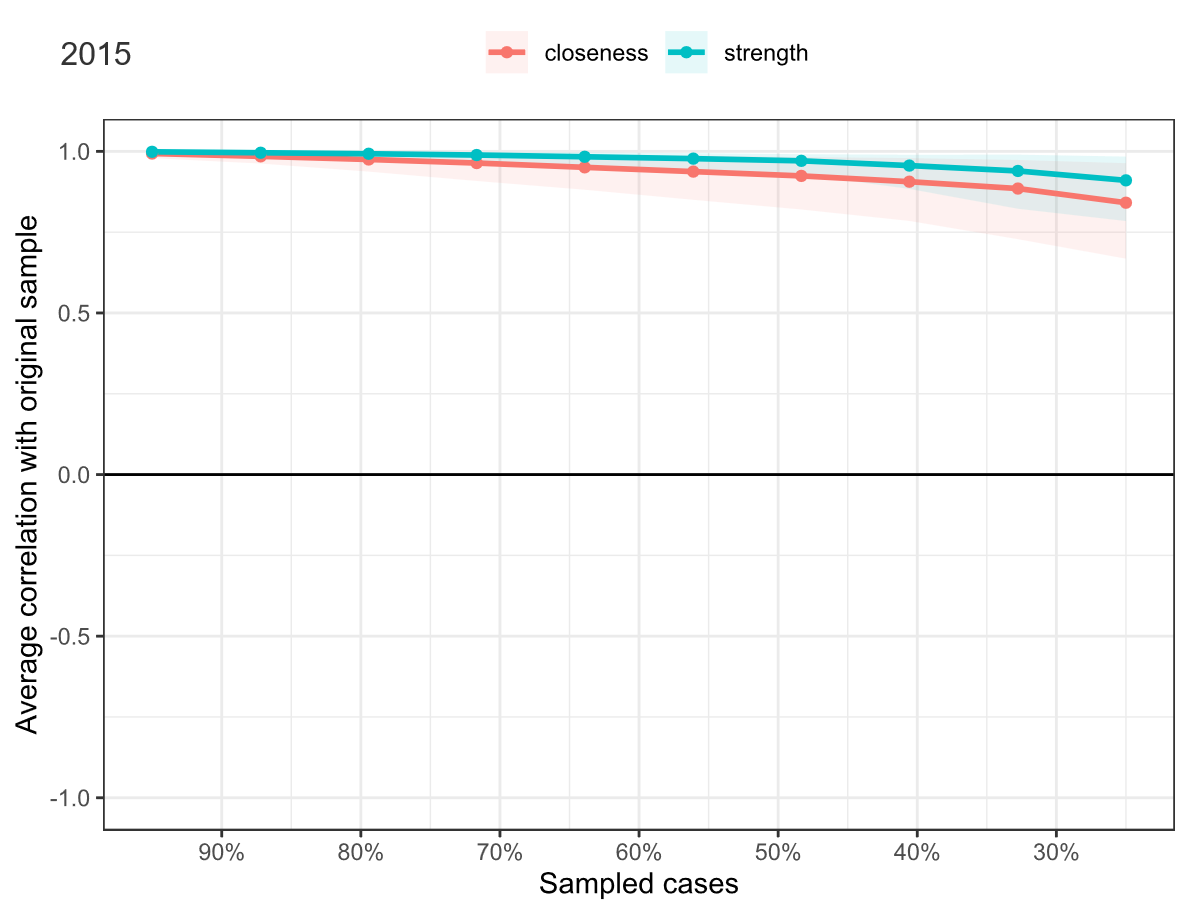

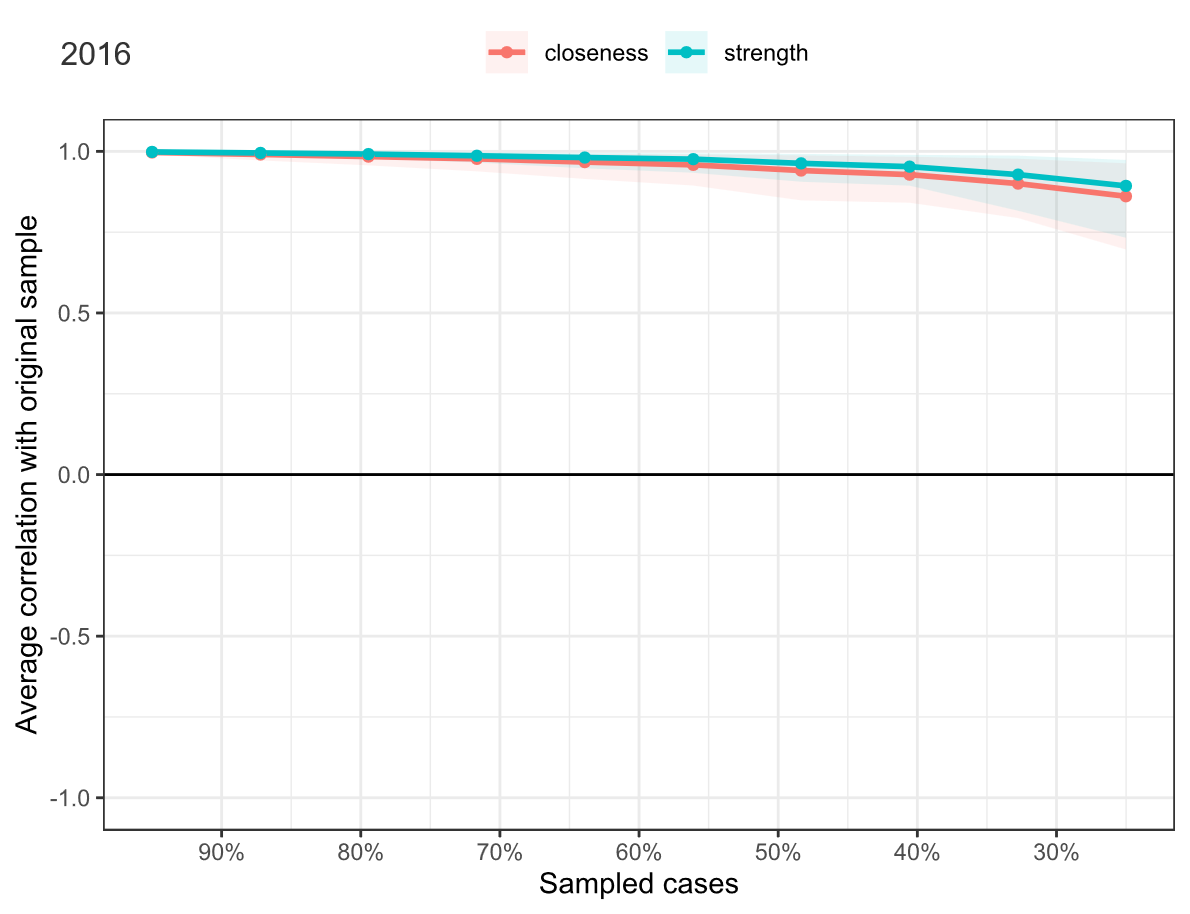

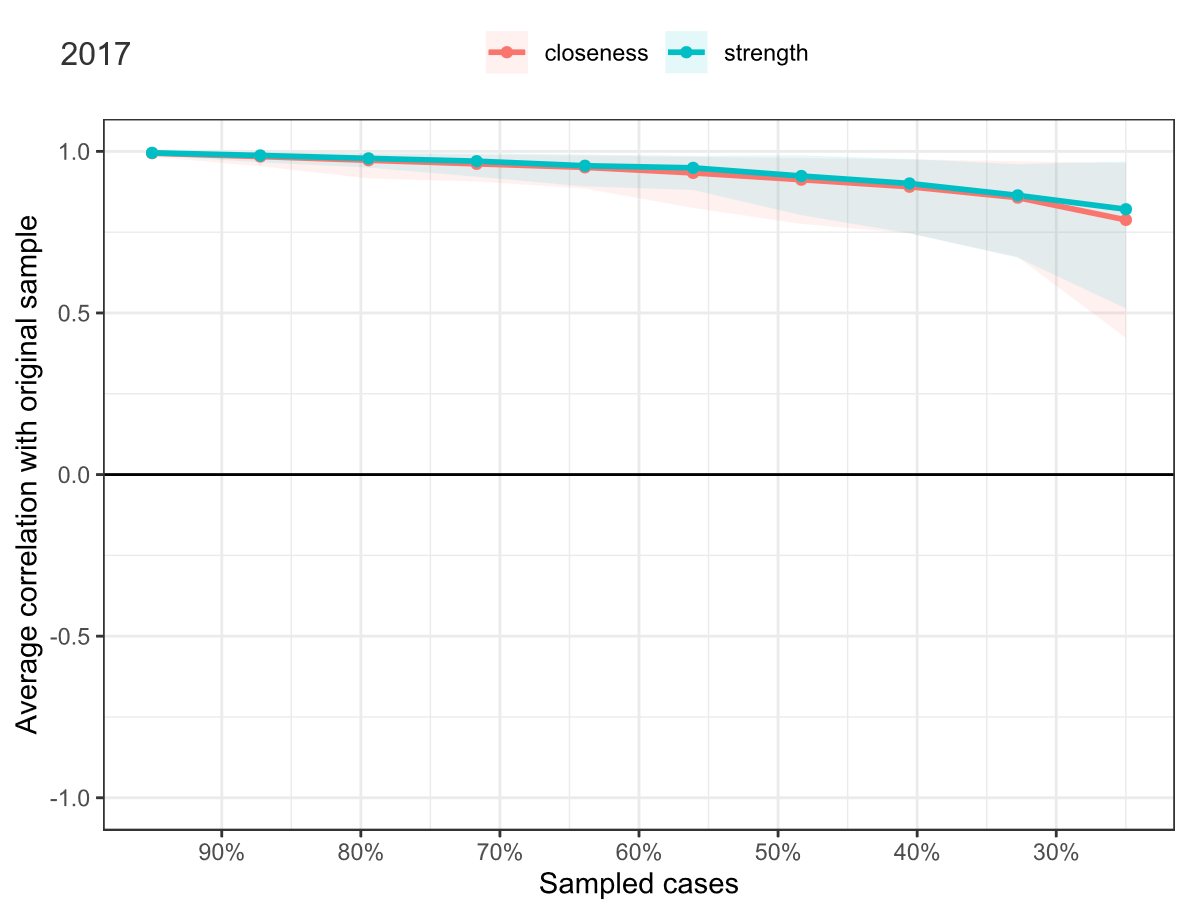

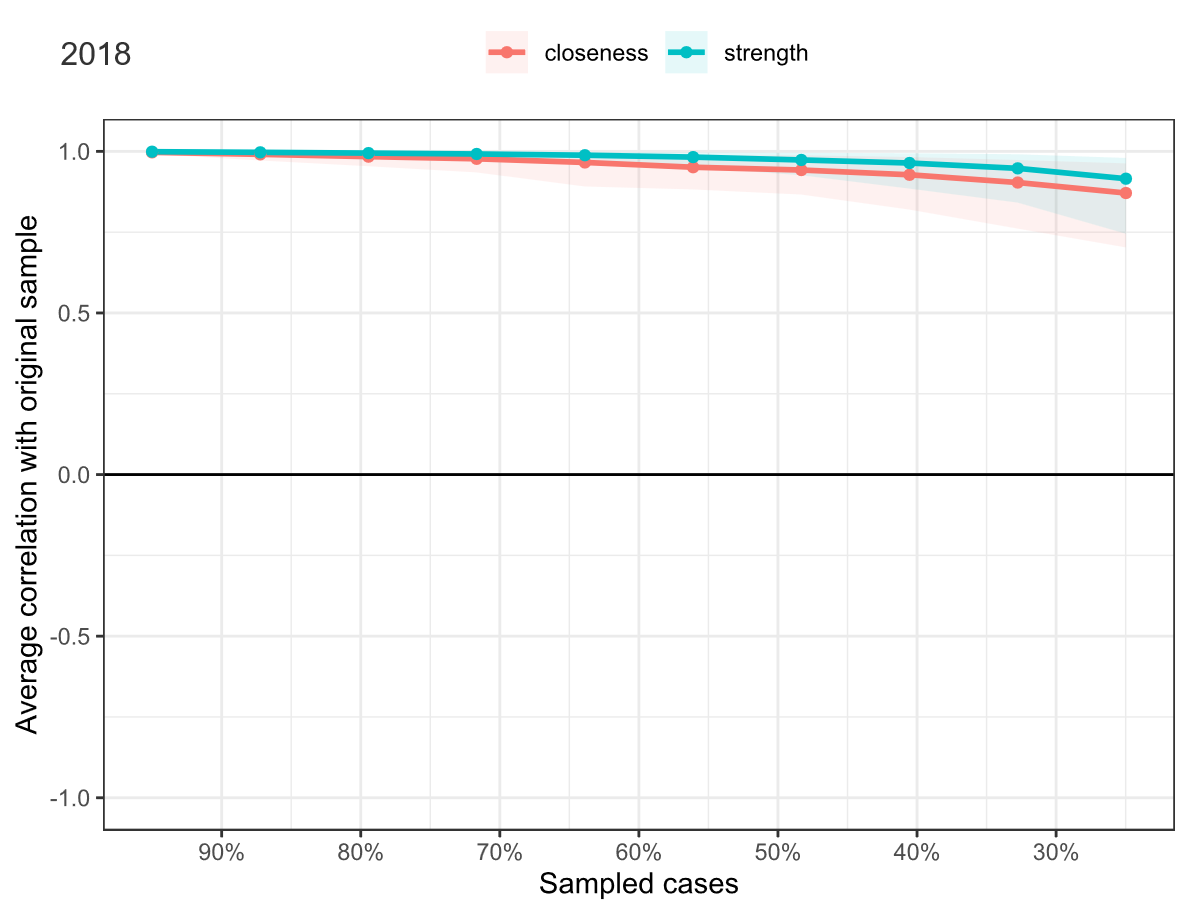

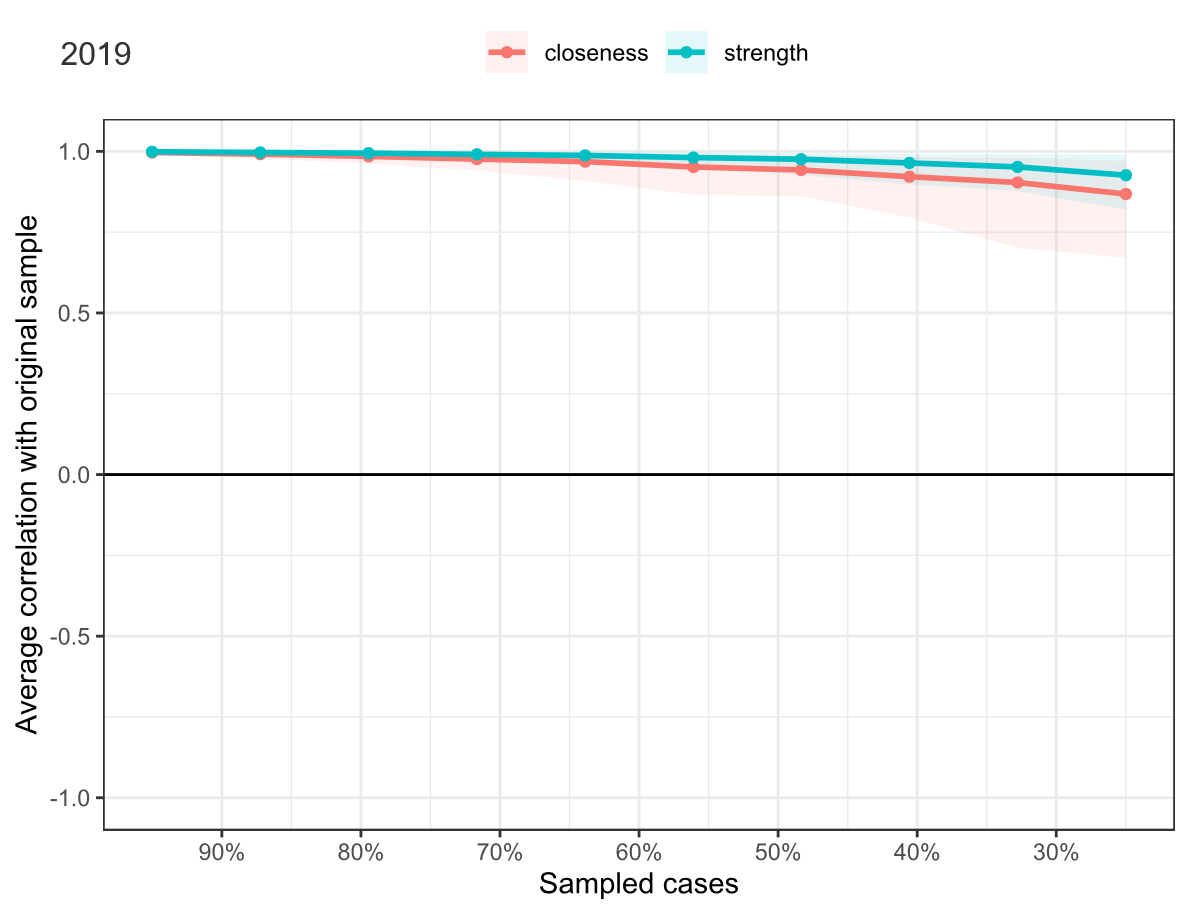

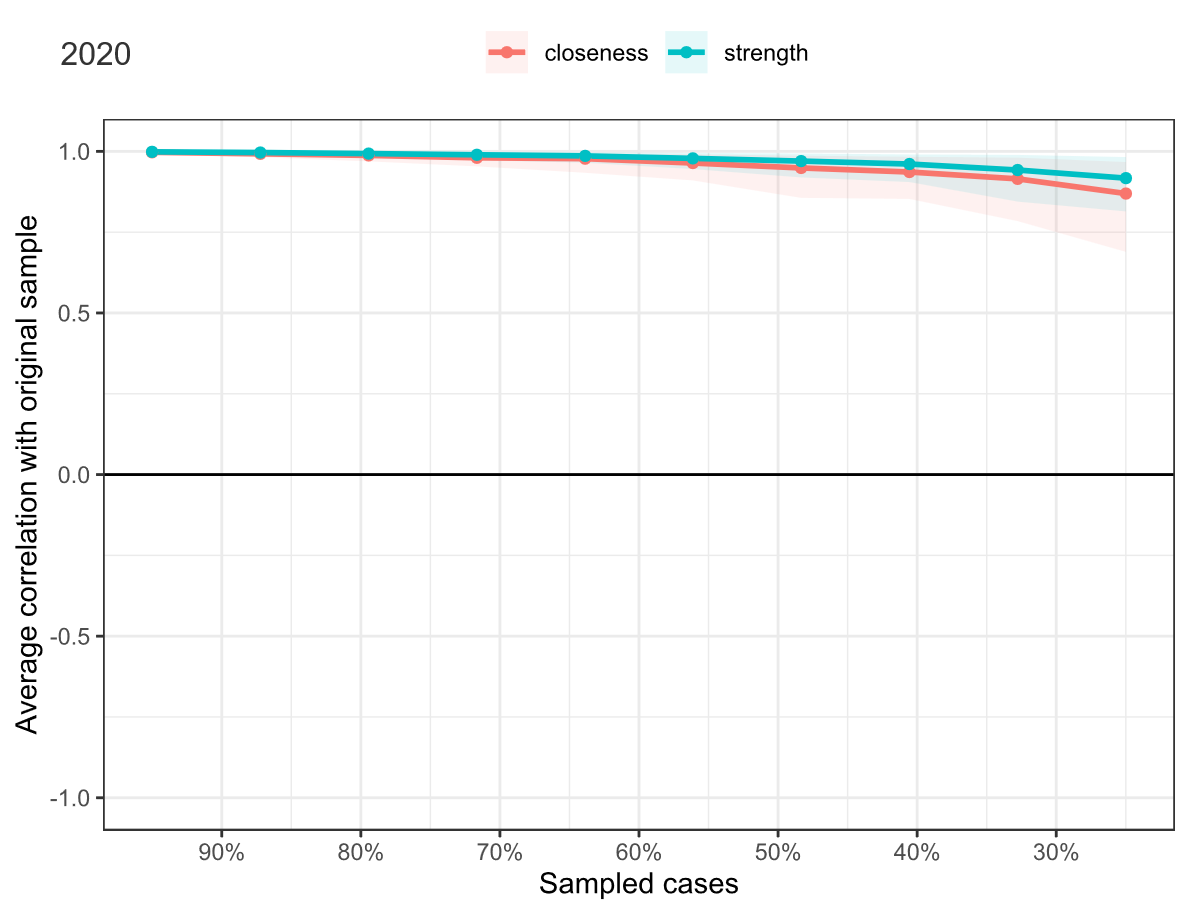

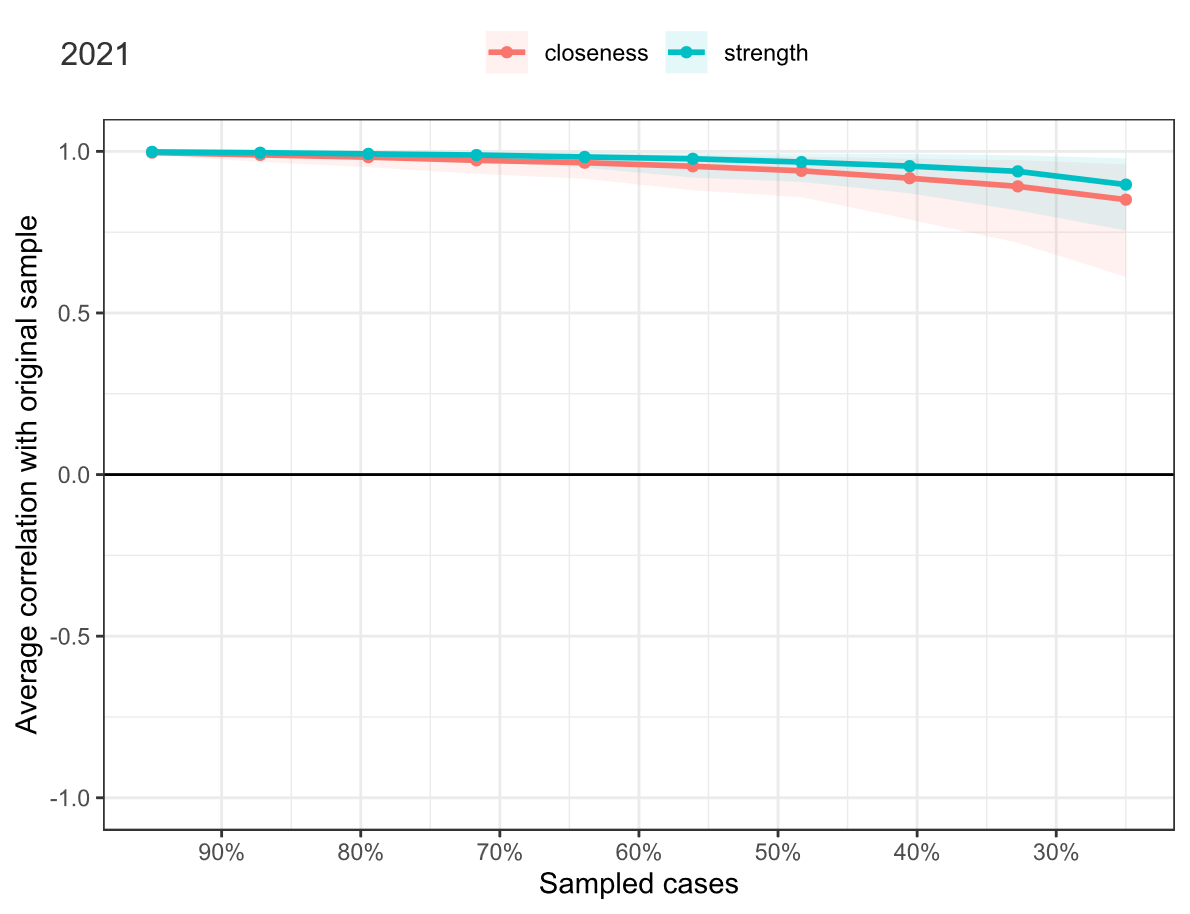

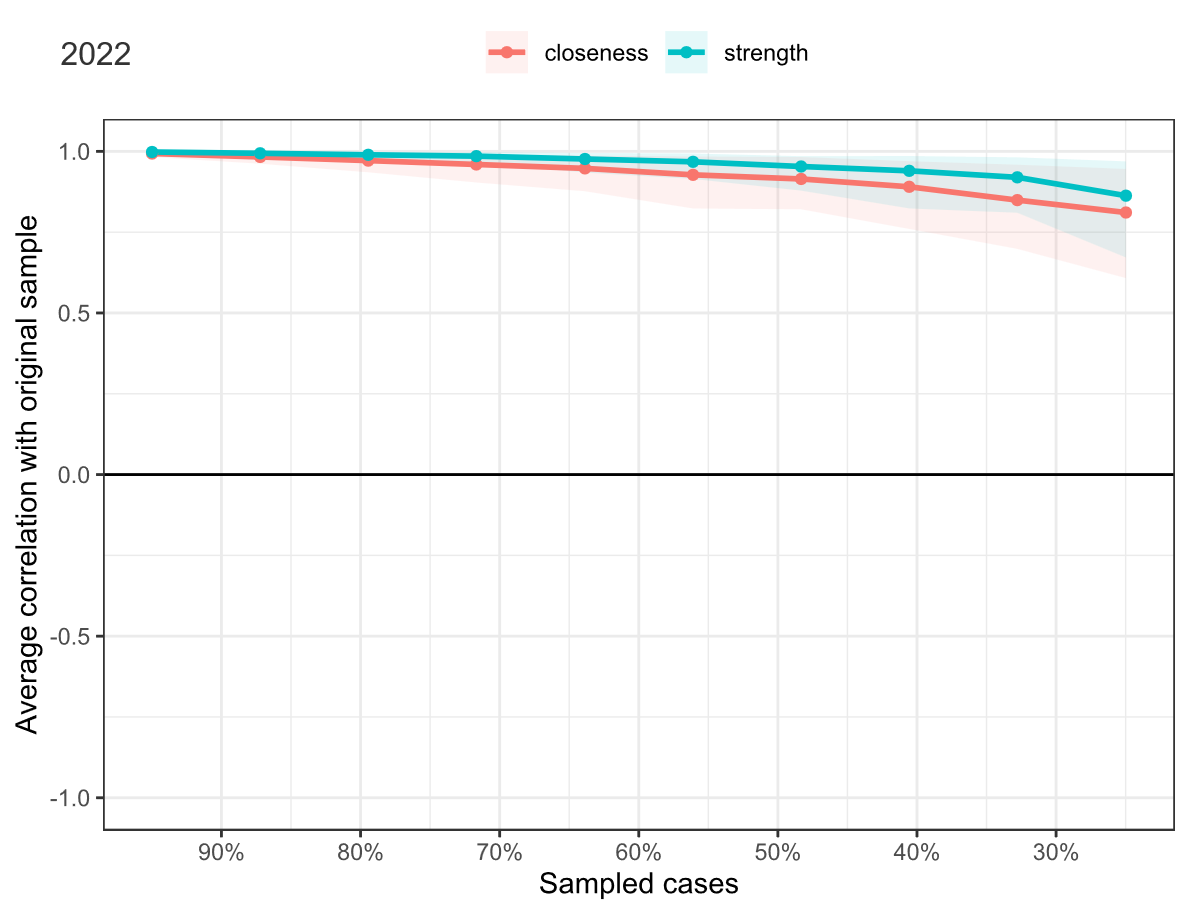

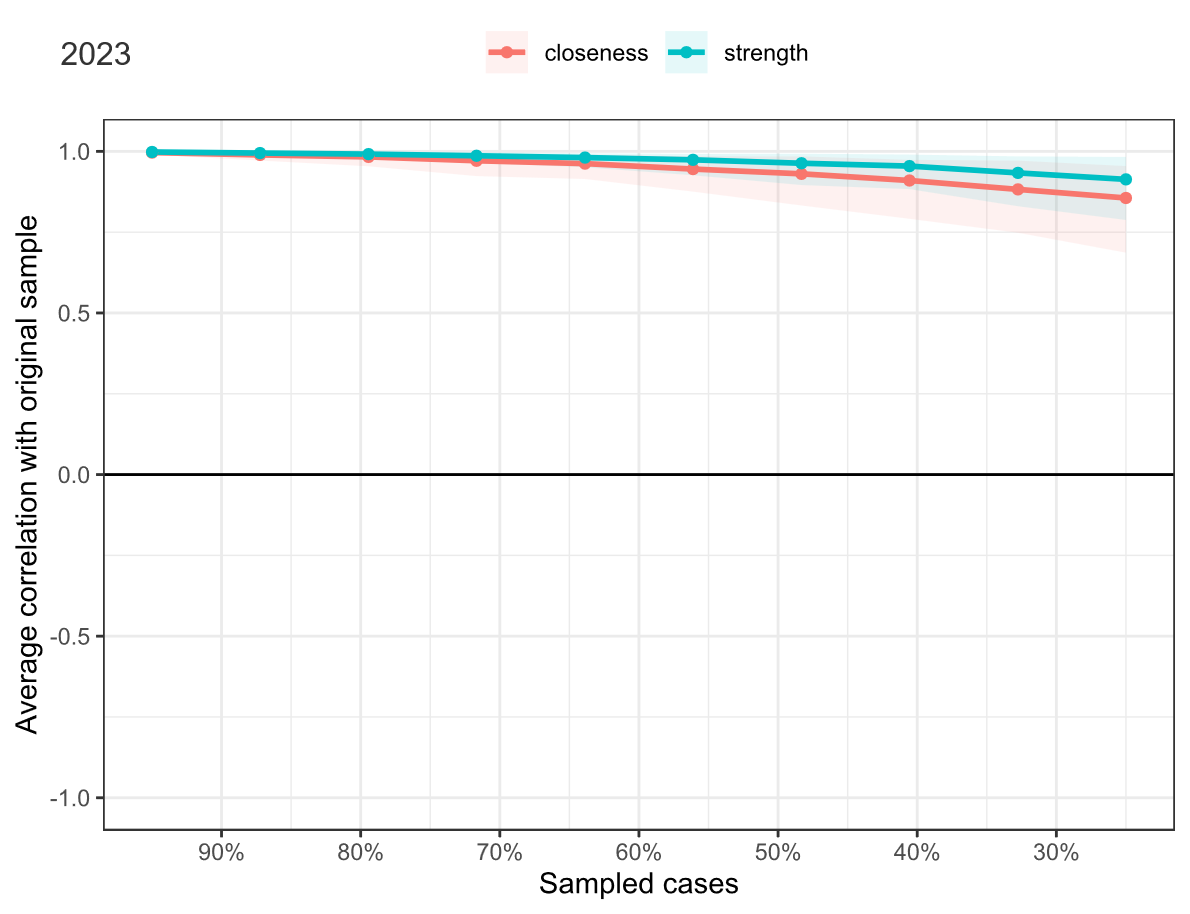

Supplement: Supplementary file 1 [file Supplementary_file_1.docx]
